# Multidrug Resistance and Plasmid Patterns of *Escherichia coli* O157 and Other *E. coli* Isolated from Diarrhoeal Stools and Surface Waters from Some Selected Sources in Zaria, Nigeria

**DOI:** 10.3390/ijerph7103831

**Published:** 2010-10-25

**Authors:** Vincent N. Chigor, Veronica J. Umoh, Stella I. Smith, Etinosa O. Igbinosa, Anthony I. Okoh

**Affiliations:** 1 Applied and Environmental Microbiology Research Group, Department of Biochemistry and Microbiology, University of Fort Hare, Private Bag X1314, Alice, 5700 Eastern Cape, South Africa; E-Mails: eigbinosa@gmail.com (E.O.I.); aokoh@ufh.ac.za (A.I.O.); 2 Department of Microbiology, Ahmadu Bello University, Funtua Road, Samaru, Zaria, 810107 Kaduna State, Nigeria; E-Mail: veroumoh@yahoo.com (V.J.U.); 3 Molecular Biology and Biotechnology Division, Nigerian Institute of Medical Research, 6, Edmund Crescent, Yaba, 2013 Lagos State, Nigeria; E-Mail: stellaismith@yahoo.com (S.I.S.)

**Keywords:** plasmid-mediated, multidrug resistance, surface water, *E. coli* O157, public health impact

## Abstract

We have assessed the prevalence of *Escherichia coli* O157 in diarrhoeal patients and surface waters from some selected sources in Zaria (Nigeria), evaluating the antibiotic susceptibility and plasmid profiles of 184 *E. coli* isolates, obtained from 228 water samples and 112 diarrhoeal stool specimens (collected from children aged <15 years), using standard methods. The detection rate of *E. coli* O157 in surface waters was 2.2% and its prevalence in children with diarrhoea was 5.4%. The most active antibiotics were gentamicin, chloramphenicol and fluoroquinolones. Seventy-nine (42.9%) of 184 *E. coli* isolates were resistant to four or more antibiotics. Multidrug resistance (MDR) was higher amongst aquatic isolates than the clinical isolates. Out of 35 MDR isolates (20 of which were O157 strains), 22 (62.9%) harboured plasmids all of which were no less than 2.1 kb in size. Amongst the 20 *E. coli* O157 strains, only seven (35.0%) contained multiple plasmids. An aquatic O157 isolate containing two plasmids was resistant to seven drugs, including ampicillin, cefuroxime, ciprofloxacin, cotrimoxazole, nalidixic acid, nitrofurantoin and tetracycline. Loss of plasmid correlated with loss of resistance to antibiotics in cured (mutant) strains selected in tetracycline (50 μg/mL)-nutrient agar plates. Our findings revealed that plasmids were prevalent in both the aquatic and clinical isolates, and suggest that the observed MDR is plasmid-mediated. The occurrence of plasmid-mediated multidrug resistant *E. coli* O157 in surface waters used as sources for drinking, recreation and fresh produce irrigation heightens public health concern.

## 1. Introduction

Antimicrobial resistance in *Enterobacteriaceae* poses a critical public health threat, especially in the developing countries [1,2]. Much of the problem has been shown to be due to the presence of transferable plasmids encoding multidrug resistance and their dissemination among different enterobacterial species [3–6].

*Escherichia coli* O157 is an important food-borne and water-borne pathogen with a worldwide distribution [7]. The first reported outbreak of *E. coli* O157 infection in the developing world occurred in 1992 in Southern Africa [8]. Outbreaks have also occurred in Central African Republic in 1996 and Cameroon, in 1997 [9]. Such outbreaks have been linked to contaminated bovine food products, contaminated drinking water and flood irrigation with water contaminated by animal faeces or surface runoff [8,10], and cattle faeces have been implicated as the major source of contamination [11].

Although not in outbreak proportions, *E. coli* O157:H7 illnesses have also been reported in Southern Nigeria [12–14]. The absence of reports on the prevalence of *E. coli* O157 and its associated infections in Northern Nigeria (where cattle is widely reared and surface waters serve as important sources of water for drinking, herd watering and fresh produce irrigation) and a heightened concern over the spread of antimicrobial resistant pathogens made this research imperative. We isolated *E. coli* from surface waters and patients (children aged <15 years) with diarrhoea in parts of Zaria, Nigeria, determined the prevalence of *E. coli* O157 strains, evaluated the antimicrobial susceptibility of all aquatic and clinical *E. coli* isolates, and screened multidrug resistant strains for plasmids.

## 2. Experimental Section

### 2.1. Isolation and Identification of E. coli and E. coli O157 Strains

The study area, as well as the collection of samples, has been described fully in our previous reports [15,16]. The stool samples were collected from diarrhoeal patients (children aged <15 years) from two hospitals, the Ahmadu Bello University (ABU) Sick Bay and Jama’a Clinic, Samaru, Zaria. *E. coli* was isolated from water samples using the Tryptic Soy Broth (TSB) enrichment and high temperature incubation method described by LeJeune *et al.* [17]. Briefly, 20 mL of each water sample was combined with equal volume of double strength TSB (Difco; Detroit, MI, USA) in a cotton-plugged 150 mL conical flask and incubated at 44.5 °C for 24 h. The resulting suspension (50 μL) was then spread plated into EMB agar. At least three discrete colonies from each water sample were randomly selected from each EMB agar plate and screened for on Sorbitol MacConkey (SMAC) agar plates (Oxoid; Basingstoke, UK). Stool specimens were cultured for *E. coli* isolation on EMB agar and incubated at 44.5 °C as described by Okeke *et al.* [18] and screened on SMAC. After incubation at 37 °C for 24 h all non-sorbitol (colourless) colonies were subjected to confirmation as *E. coli* isolates by Gram-staining and standard biochemical tests and recorded as presumptive *E. coli* O157. Confirmation of *E. coli* O157 was done by testing for agglutination with *E. coli* O157 antisera (Oxoid). EHEC strain EDL 933 was used as positive control.

### 2.2. Antimicrobial Susceptibility Testing

All *E. coli* isolates were tested for antimicrobial resistance using the method of Bauer *et al.* [19] on standard antibiotic discs (PS003G-VE, Polytes Laboratories, Enugu, Nigeria) using the following antibiotics: ampicillin (25 μg), cefuroxime (20 μg), chloramphenicol (10 μg), ciprofloxacin (5 μg), cotrimoxazole (50 μg), gentamicin (10 μg), nalidixic acid (30 μg), nitrofurantoin (100 μg), norfloxacin (10 μg) and tetracycline (50 μg). *E. coli* ATCC 25922 was used as control organism, and the results were interpreted using the Clinical and Laboratory Standards Institute (CLSI) criteria [20].

### 2.3. Plasmid Extraction, Screening and Curing

Extraction of plasmid DNA was done using the alkaline lysis method of Birnboim and Doly [21]. A 0.8% (w/v) agarose gel was used. Crude DNA extract solutions were subjected to electrophoresis using a horizontal apparatus—the Minnie submarine agarose gel unit (HE33; Hoefer, San Francisco, CA, USA) and a constant voltage (60V) power source (P500B; Sigma, St. Louis, MO, USA) for 2–3 h as described Meyers *et al.* [22]. Gels were observed using a 302 nm UV trans-illuminator (Vilber Lourmat) and photographed with a Polaroid camera. The sizes of the plasmids were estimated as described elsewhere [22,23]. The sodium dodecyl sulphate (SDS) treatment method of Tomoeda *et al.* [24] was used for plasmid curing. Tetracycline-resistant, plasmid-containing *E. coli* isolates were chosen and subjected to curing and antibiotic susceptibility testing. After the curing experiments, the tetracycline-susceptible, lactose-fermenting and indole-positive colonies found to have lost their plasmid(s) were considered cured (mutant) *E. coli* strains, transferred onto nutrient agar slants, incubated at 37 °C for 24 h, and subjected to antibiotic susceptibility testing as already described, using the same set of antibiotics.

## 3. Results and Discussion

### 3.1. Prevalence of E. coli O157and Antibiotic Susceptibility of Isolates

A total of 184 *E. coli* isolates were obtained from 228 water samples and 112 diarrhoeal stool specimens. Twenty-one (11.4%) of these 184 isolates were found to be non-sorbitol-fermenting, out of which 20 (95.2%) were confirmed to be *E. coli* O157 by slide agglutination. Table 1 shows the prevalence of *Escherichia coli* O157 in surface waters and among diarrhoeal patients. Only five of the 228 water samples were positive for *E. coli* O157, corresponding to an isolation rate of 2.2 %. Only six of the 112 stool specimens gave positive cultures of *E. coli* O157, and hence a prevalence of 5.4%.

Table 2 shows the antimicrobial susceptibility patterns of 184 *E. coli*, isolated from clinical and water samples, using 10 antibiotics. Gentamicin, norfloxacin and chloramphenicol were the most active. While all of the clinical isolates were sensitive to both gentamicin and norfloxacin, and only 6.8% was resistant to chloramphenicol, only 2.1%, 4.9% and 5.4% of the aquatic isolates were, resistant to the three antibiotics, respectively. Also, of the 184 isolates, 154 (83.7%) and 123 (66.8%) were resistant to ampicillin and tetracycline, respectively, implying that ampicillin and tetracycline were the least active.

Table 3 reveals the multidrug resistance (MDR) patterns of the 184 *E. coli* isolates. MDR was higher amongst aquatic isolates than the clinical isolates. While 58 (60.4 %) of the aquatic strains showed resistance to ampicillin, tetracycline and nitrofurantoin, only 22 (25 %) of the clinical isolates gave similar results. Overall, higher numbers of resistant isolates were identified among the aquatic isolates. The highest levels of MDR observed were in two aquatic isolates: one was resistant to eight antibiotics and another to nine of the 10 antibiotics tested, including ampicillin, cefuroxime, chloramphenicol, ciprofloxacin, cotrimoxazole, gentamicin, nalidixic acid, norfloxacin and tetracycline.

Table 4 shows that amongst the 20 *E. coli* O157 isolates (14 aquatic and six clinical strains), the most active antibiotics were the fluoroquinolones and chloramphenicol. While none (0.0 %) was resistant to norfloxacin, three (15 %) showed intermediate susceptibility and 17 (85 %) were sensitive. For ciprofloxacin, while 18 (90%) were sensitive, only 2 (10%) were sensitive. Seventeen (85 %) were sensitive to chloramphenicol. Further, 16 (80 %) of the *E. coli* O157 isolates were sensitive to each of cotrimoxazole and gentamicin. All (100 %) of the six clinical O157 isolates were sensitive to ciprofloxacin and chloramphenicol, while five (83.3 %) and three (50 %) were resistant to tetracycline and ampicillin respectively. Amongst the 14 aquatic isolates, while 10 (71.4%) were resistant to ampicillin and tetracycline, nine (64.3%) were resistant to nitrofurantoin and 13 (93%) to chloramphenicol.

### 3.2. Plasmid Studies

Thirty-five multidrug-resistant *E. coli* isolates were screened for plasmids. This consisted of 23 aquatic and 12 clinical isolates, grouped further into 20 *E. coli* O157 strains and 15 non-O157 strains. Table 5 shows that 22 (62.9%), of the 35, haboured plasmids (>2.1 Kb). The table further reveals that out of the 22 plasmid-containing isolates, only seven (JC17, JC15, LR 11, BS12, AS04, FD08 and FD07), marked with asterisks (*) are *E. coli* O157 strains. All (100%) of the 15 non-O157 isolates contained plasmids. The resistance level observed in this 22 ranged from two-drug resistance (Isolate JC17) to nine-drug resistance (Isolate AS03).

All (100%) of the 22 plasmid-containing isolates were resistant to tetracycline, while only 10 (45.5%) were resistant to ciprofloxacin. In all, eight isolates contained two or more plasmids, giving a prevalence of 22.9%. Each of the isolates bearing multiple plasmids was resistant to at least four antibiotics. Interestingly, Isolate AS03 which showed resistance to nine different antibiotics contained only one plasmid.

Amongst the 20 *E. coli* O157 isolates, the DNA fingerprints revealed that only seven (35%) contained plasmids, out of which three (15%) contained more than one plasmid. One of the aquatic isolates (FD07) containing two plasmids was resistant to seven drugs including ampicillin, cefuroxime, ciprofloxacin, cotrimoxazole, nalidixic acid, nitrofurantoin and tetracycline. No clinical *E. coli* O157 isolate contained multiple plasmids.

Plasmid curing experiments were carried out to determine if the drug resistance observed in this study was plasmid-mediated. No plasmid bands were observed after the electrophoretic separation of crude DNA extracts from seven tetracycline-susceptible mutants. Loss of plasmids correlated with loss of resistance to antibiotics in cured strains selected on tetracycline (50 μg/mL)-nutrient agar plates. Loss of resistance to multiple antibiotics was also observed. Six (86%) of the tetracycline-susceptible mutant showed sensitivity to tetracycline and ampicillin.

### 3.3. Discussion

The detection of *E. coli* O157 at relatively high frequencies in these waters (stream, 1.7%; dam, 2.7%; river, 2.1%) and its occurrence heighten public health concern about these surface waters that are sources of raw water treated for drinking, recreation, irrigation and herd watering [25]. Kubanni River, used by local farmers for the irrigation of commercial crops (tomatoes, lettuce, cabbage, onions, spinach, sugarcane etc), has been reported as unfit for fresh produce irrigation [26]. The 5.4% prevalence of *E. coli* O157 in children with diarrhoea suggests that the epidemiological situation in Nigeria had remained the same since 1994 [14].

Comparison of antibiotic susceptibility in clinical and water isolates showed that higher levels of resistance existed in the aquatic isolates. This agrees with the findings of Idika [25], who studied *Vibrio cholerae* isolates during an outbreak of cholera in Lagos in 1997 and reported that the isolates from water were resistant to tetracycline and gentamicin whereas clinical isolates were susceptible. Our findings which are not enough to suggest any epidemiological link between the aquatic and clinical strains concur with that of Okoh and Igbinosa [26]. They studied antibiotic susceptibility patterns of the *Vibrio* isolates as well as the distribution of antibiotic resistance genes in the isolates and found that previous use of antibiotics in earlier outbreaks may be, in part, responsible for the extensive increase in antibiotics resistance. It is unknown whether the isolates responsible for earlier and recent epidemics are of the same clonal origin. The association between the development of resistance to ampicillin and tetracycline with large-scale use of antibiotics for treatment and prophylaxis of diarrhoea is well recognized Idika [25]. Ash *et al.* [27] also reported high levels of resistance in gram-negative bacteria in rivers in the United States.

All (100%) of the 22 plasmid-containing isolates were resistant to tetracycline. This is worrisome considering that tetracycline is a first line drug in Nigeria, and as in most developing countries, people with gastrointestinal infections readily purchase it across the counter for self-medication.

From the results, it appears that plasmids are ubiquitous in bacterial population in the waters. Different plasmids (2–3) often coexisted in the same host cell. This agrees with the finding of Fujita *et al.* [28] who isolated 45 antibiotic resistant bacteria from wastewater samples and detected 31 plasmids in 14 of 45 antibiotic resistant strains with 10 carrying multiple (2–4) plasmids. The demonstration of high-molecular-weight plasmids in the isolates, and the finding that the expressed multi-drug resistance (MDR) was plasmid-mediated is significant. McPherson and Gealt [3] showed that several enteric bacterial strains isolated from wastewater samples and possessing both antibiotic resistance and high molecular-weight plasmids could transfer their resistance to other recipient bacteria. The transmissibility of resistance (R)-genes and plasmids poses public health risk, considering the vast potential of hosts presented by microbial populations in the gut and water environment. This risk could be heightened if R-genes are disseminated across geographic borders by travellers or by a river continuum, like Kubanni that traverses different communities within the study area.

Mutant cells obtained by SDS treatment of tetracycline-resistant isolates were susceptible to tetracycline suggesting that a part or all of their resistance genes must have been deleted by SDS action. Loss of plasmids correlated with loss of resistance to ampicillin, chloramphenicol, ciprofloxacin, norfloxacin and tetracycline in 6 (86%) of the mutants. It therefore appears that the observed multi-drug resistance to these antibiotics was plasmid-mediated. This agrees with previous findings.

Ash *et al.* [27] had in a study of the antibiotic resistance of gram-negative bacteria in rivers in the United States demonstrated that resistance to ampicillin and other drugs (including ciprofloxacin, tetracycline, chloramphenicol, kanamycin and streptomycin) was plasmid-mediated. Interestingly, two mutants now resistant to cotrimoxazole originated from wild-type *E. coli* O157 isolates that were sensitive to the same drug. This could be attributed to possible mutation affecting chromosomal genes in the course of the curing experiment [29].

## 4. Conclusions

Pathogenic *Escherichia coli* isolated from these surface water and clinical sources were found to be resistant to tetracycline, cotrimoxazole, ampicillin and other commonly used antibiotics. Higher levels of resistance were observed in aquatic isolates. Multidrug resistance and plasmid were observed in both aquatic and clinical isolates. Loss of plasmids due to treatment with sodium dodecyl-sulphate correlated with loss of resistance to antibiotics, suggesting that the observed multidrug resistance was plasmid-mediated. The occurrence of plasmid-mediated multidrug resistance in bacteria in these surface waters heightens the public health concern. The study showed a need for a continuous pollution monitoring programme of the surface waters in Nigeria.

## Figures and Tables

**Table 1.** Prevalence of *Escherichia coli* O157 in Surface waters and among Diarrhoeal Patients in Zaria, Nigeria.

**Sample**	**Source**	**Number (%) of Collected Samples**	**Number (%) positive for*E. coli*O157**
Water	Samaru Stream	58 (25.4)	1 (1.7)
	Kubanni River	96 (42.1)	2 (2.1)
	ABU Dam	74 (32.5)	2 (2.7)
	**Total**	**228 (100.0)**	**5 (2.2)**

Diarrhoeal Stool	ABU Sick Bay	35 (31.3)	1 (2.9)
	Jama’a Clinic	77 (68.7)	5 (6.5)
	**Total**	**112 (100.0)**	**6 (5.4)**

**Table 2.** Antimicrobial Susceptibility Pattern of *E .coli* Isolated from Clinical and Water Samples.

**S/N**	**Antimicrobial Agent**	**Symbol**	**Disc Content (μg)**	**Susceptibility**						
				**Clinical Isolates (n = 88)**			**Aquatic Isolates (n = 96)**			**Resistant Isolates Combined (n= 184) No. (%)**
				**R No. (%)**	**I No. (%)**	**S No. (%)**	**R No. (%)**	**I No. (%)**	**S No. (%)**	
1	Ampicillin	Amp	25	71 (80.7)	14 (15.9)	3 (3.4)	83 (86.5)	5 (5.2)	8 (8.3)	154 (83.7)
2	Cefuroxime	Cef	20	28 (31.8)	9 (10.2)	51 (58.0)	71 (74.0)	11 (11.4)	14 (14.6)	99 (53.8)
3	Chloramphenicol	Chl	10	6 (6.8)	0 (0.0)	82 (93.2)	4 (4.2)	1 (1.0)	91 (94.8)	10 (5.4)
4	Ciprofloxacin	Cip	5	17 (19.3)	3 (3.4)	68 (77.3)	11 (11.5)	2 (2.1)	83 (86.4)	28 (15.2)
5	Cotrimoxazole	Cot	50	9 (10.2)	2 (2.3)	77 87.5	14 (14.6)	2 (2.1)	80 (83.3)	23 (12.5)
6	Gentamicin	Gen	10	0.(0.0)	0 (0.0)	88 (100)	2 (2.1)	7 (7.3)	87 (90.6)	2 (1.1)
7	Nalidixic Acid	Nal	30	48 (54.6)	9 (10.2)	31 (35.2)	68 (70.8)	9 (9.4)	19 (19.8)	116 (63.0)
8	Nitrofurantoin	Nit	100	45 (51.2)	6 (6.8)	37 (42.1)	76 (79.1)	4 (4.2)	16 (16.7)	121 (65.8)
9	Norfloxacin	Nor	10	0 (0.0)	6 (6.8)	82 (93.2)	9 (9.4)	5 (5.2)	82 (85.4)	9 (4.9)
10	Tetracycline	Tet	50	54 (61.4)	5 (5.6)	29 (33.0)	69 (71.9)	7 (7.3)	20 (20.8)	123 (66.8)

Legend: R, resistant; I, intermediate; S, sensitive.

**Table 3.** Resistance Patterns of Aquatic and Clinical in *E. coli* Isolates.

**Number of Antibiotics**	Combination of Antibiotics*	**Aquatic Isolates (n = 96)**	**Clinical Isolates (n = 88)**	**Total**	***E. coli*O157**

				**(n = 184)**	**Isolates (n = 20)**
2	Amp, Cef	59(61.50)	8(9.1)	67(36.4)	12(60.0)
2	Amp, Cot	13(13.5)	4(4.5)	17(9.2)	2(10.0)
2	Amp, Tet	63(65.6)	39(44.3)	102(55.4)	13(65.0)
2	Cot, Tet	10(10.4)	5(5.7)	15(8.2)	2(65.0)
3	Amp,Tet, Chl	5(5.2)	2(2.3)	7(3.8)	1(0.0)
3	Amp, Cot, Nit	9(9.4)	3(3.4)	12(6.5)	1(5.0)
3	Amp, Tet, Cot	9(9.4)	4(4.5)	13(7.1)	1(5.0)
3	Amp, Tet, Nit	58(60.4)	22(25.0)	80(43.5)	9(45.0)
4	Amp, Tet, Nal, Nit	50(52.1)	21(23.4)	71(38.6)	9(45.0)
5	Amp, Tet, Cip, Nal, Nit	8(8.3)	5(5.7)	13(7.1)	1(5.0)
6	Amp, Tet, Cip, Cot, Nal, Nit	6(6.3)	3(3.4)	9(4.9)	1(5.0)
7	Amp, Tet, Cip, Cef, Cot, Nal, Nit	2(2.1)	1(1.1)	3(1.6)	1(5.0)
8	Amp, Tet, Cip,Cef, Cot,Chl, Nal, Nit	1(1.0)	0(0.0)	1(0.5)	0(0.0)
9	Amp, Tet, Cip,Cef, Cot,Chl, Gen, Nal, Nor	1(1.0)	0(0.0)	1(0.5)	0(0.0)

Symbols: Amp, ampicillin; Cef, cefuroxime; Cot, cotrimoxazole; Tet, tetracycline; Chl, chloramphenicol; Nit, nitrofurantoin; Nal, nalidixic acid; Cip, ciprofloxacin; Gen, gentamicin; Nor, norfloxacin.

**Table 4.** Antimicrobial susceptibility pattern of *E. coli* O157 strains isolated from clinical and water samples.

**S/N**	**Antimicrobial Agent**	**Symbol**	**Disc Content (μg)**	**Susceptibility (n = 20)**		
				**Resistant No. (%)**	**Intermediate No. (%)**	**Sensitive No. (%)**
1	Ampicillin	Amp	25	13 (65.0)	5 (25.0)	2 (10.0)
2	Cefuroxime	Cef	20	10 (50.0)	3 (15.0)	7 (35.0)
3	Chloramphenicol	Chl	10	1 (5.0)	2 (10.0)	17 (85.0)
4	Ciprofloxacin	Cip	5	2 (10.0)	0 (0.0)	18 (90.0)
5	Cotrimoxazole	Cot	50	3 (15.0)	1 (5.0)	16 (80.0)
6	Gentamicin	Gen	10	2 (10.0)	2 (10.0)	16 (80.0)
7	Nalidixic Acid	Nal	30	11 (55.0)	2 (10.0)	7 (35.0)
8	Nitrofurantoin	Nit	100	9 (45.0)	3 (15.0)	8 (40.0)
9	Norfloxacin	Nor	10	0 (0.0)	3 (15.0)	17 (85.0)
10	Tetracycline	Tet	50	15 (75.0)	2 (10.0)	3 (15.0)

**Table 5.** Plasmid Profile and Multidrug Resistance Patterns of Plasmid-Containing *E. coli* Isolates.

***E. coli*isolates**	**Number of plasmids**	**Antibiotics to which Isolates were Resistant**	
		**Number**	Combinations**
JC17*	1	2	Amp, Tet
JC15*	1	3	Amp, Tet, Cot
LR11*	2	4	Amp, Tet, Nit, Nal
JC11	1	4	Amp, Tet, Nit, Nal
BS12*	1	4	Amp, Tet, Nit, Cef
JC28	1	4	Amp, Tet, Cip, Cot
AS04*	1	5	Amp, Tet, Nit, Nal, Cef,
FD08*	2	5	Amp, Tet, Nit, Nal, Cef
AS06	1	5	Amp, Tet, Nit, Nal, Cef
SB06	2	5	Amp, Tet, Nit, Nal, Cip
SB13	1	5	Amp, Tet, Nit, Nal, Cip
KR10	1	5	Amp, Tet, Nit, Nal, Cot
HR09	1	5	Cef, Tet, Nit, Nal, Cot
SB04	2	6	Amp, Tet, Nit, Nal, Cip, Cot
IR10	1	6	Amp, Tet, Nit, Nal, Cef, Cot
JC13	1	6	Amp, Tet, Cef, Cip, Cot, Chl
FD07*	2	7	Amp, Tet, Nit, Nal, Cef, Cip, Cot
SB09	2	7	Amp, Tet, Nit, Nal, Cef, Cip, Cot
JR10	3	7	Amp, Tet, Nit, Nal, Cef, Cip, Cot
BS06	1	7	Amp, Tet, Nit, Nal, Cef, Cot, Chl
KR08	2	8	Amp, Tet, Nit, Nal, Cef, Cip, Cot, Chl
AS03	1	9	Amp, Tet, Nit, Nal, Cip, Cot, Chl, Gen, Nor

Legend:

* *E. coli* O157 strains;

** Amp, ampicllin; Cef, cefuroxime; Cot, cotrimoxazole; Tet, tetracycline; Chl, chloramphenicol; Nit, nitrofurantoin; Nal, nalidixic acid; Cip, ciprofloxacin; Gen, gentamicin; Nor, norfloxacin.
